# Ten simple rules for short and swift presentations

**DOI:** 10.1371/journal.pcbi.1005373

**Published:** 2017-03-30

**Authors:** Christopher J. Lortie

**Affiliations:** 1 Department of Biology, York University, Toronto, Ontario, Canada; 2 National Center for Ecological Analysis and Synthesis, UCSB, Santa Barbara, California, United States of America

## Preamble

Scientific communication is an independent research domain and has become a fundamental component of most scientific discourse and all public outreach. It now comprises a set of critical activities for many research programs [1, 2], including those that directly influence global and human health [3]. Scientific communication has evolved because it does not have to happen only at the final stages of a research endeavor but can be used to engage the public to fund the research (https://scifundchallenge.org), participate in the data collection (http://www.audubon.org/conservation/science/christmas-bird-count), share or crowd source the code and analyses (https://github.com), and process the evidence (https://www.zooniverse.org). Unfortunately, scientific progress in some fields such as climate change has outpaced our capacity to effectively communicate and contextualize findings for the public [4]. To mitigate this shortcoming, resources specific to scientists have been developed [5–8]. Boot-camp training workshops are now also offered (i.e., American Institute of Biological Sciences [AIBS]), and discussion of how academics use brief communications, such as social media tools, is present within the primary research literature [9–11]. An interesting related opportunity has emerged that, in some respects, bridges the gap between lengthy, detailed presentations of scientific findings and “sound bites” such as headlines or short press releases appropriate for media reporting: very short, swift presentations. Admittedly, these talks are in essence sound bites, too, but with more depth and thus requiring special consideration in terms of how to best leverage their potential [12]. These shorter presentations are commonly directed both to peers at scientific conferences and to the general public at in-person events and online. This format is particularly suited to online dissemination and sharing through YouTube, with most major scientific institutions and organizations administering channels of curated content. Many major scientific conventions include offerings of rapid-fire format talks—at first to communicate meta-science but now also to share primary research findings. The specific guidelines vary, but the slide deck is often limited by a set number of slides, or the presentation is limited by very strict, short time constraints (such as found with lightning talks). In addition, the slides can be set to rapidly autoadvance, for instance, with PechaKucha presentations. These presentation formats are also organized into open, public series and feature involvement from experts in many disciplines on numerous topics, including science. Succinct prose is thus a critical element in communicating science using these presentation formats. On a cautionary note, reducing much longer talks to these shorter formats is likely not the most effective strategy because shorter total presentation times coupled with rapid pacing can dramatically influence the scope and depth of the material. Best practices for scientific communication certainly apply to these talks, but specific strategies are nonetheless needed. For instance, as a general rule-of-thumb, talks prepared for a more general public audience should emphasize the implications of the science and use direct, natural language and visual analogies (instead of necessarily always showing complex evidence or primary data). Talks for scientific colleagues must also embrace parsimony but can accommodate more technical language depending on the specific audience, more direct evidence, and some data visualization that highlights complexity appropriately.

Effective oral prose is not dissimilar from effective writing. Depending on the literary theory and school of criticism that one subscribes, concepts such as “lightness, quickness, exactitude, visibility, multiplicity,” and also consistency in writing [13] similarly apply to rapid presentations. The simple rules for making good presentations also apply [14], but short, swift presentations provide both novel challenges and unique communication opportunities. The pace is rapid, providing very limited time for the audience to read or process an individual slide. Thus, Rule 8 from a previous ten simple rules for oral presentations, “use visuals sparingly but effectively” [14], is a best practice for this context. If the slide deck autoadvances, the speaker must perfectly time delivery, and thus, preparation relative to a longer, less structured talk differs. The net time is significantly reduced from even most typical conference oral presentations, thereby limiting the potential scope of coverage of a topic and depth. This suggests a further reduction in the number of take-home messages suggested for longer presentations (i.e., Rule 4 from a previous set of rules suggests three points should be retained, whilst here it is likely fewer, such as one) [14]. These challenges can become benefits if handled effectively. A swift tempo engenders enthusiasm, energy, and the expectation that a bird’s-eye view of a topic will be provided to, quite literally, “get the audience up to speed” on the salient issues. In the spirit of light, quick, and exact (but without the lazy dog), here are ten simple rules for presentation formats that do not wait for the speaker. A slide deck and a video of these suggestions are also available (http://bit.ly/short-swift).

## Rule 1: Plan a clear story

Avoid detours, tangents, or side anecdotes. Amusing anecdotes by accomplished speakers can be compelling, useful tools to engage and connect the speaker to the audience emotionally. In longer talks, they can also serve as a reprieve from detail-laden or inaccessible issues, and anecdotes can reframe the science into more general contexts. In a short, brisk talk, however, immediacy is paramount, and tangents are best avoided. Prepare one primary message for the audience. A total of 20 slides or 5–6 minutes, for instance, do not leave sufficient room for a story within a story. A clear story can captivate and illuminate, but a planned story is more likely to do both.

## Rule 2: Provide only one major point per slide

You have a story to tell with very limited time. Ensure each slide is a meaningful step. Some of the slides can be used to support a difficult step taken by clarifying the briefest of introductions on the previous slide. This reinforcement ensures that the audience is sufficiently informed to move forward with you on the following slide. Build on your clear story (Rule 1) one step at a time. Balance support and advancement appropriately.

## Rule 3: Limit use of text

It is much quicker for you to directly state the purpose of a given slide. Nonetheless, parsimonious use of text can assist the audience in scanning each slide for meaning and relevance. Treat the slides like scientific figures—“captions are not optimal” [15] but can be powerful aids if they do not detract from the visuals. An alternative approach is to show a visual/figure on a full slide, maximized for viewing, and use the subsequent slide for a single, brief sentence stating the finding or implication. This has added value in that it provides the speaker with more time to explain the findings and mimics a rapid but effective show-and-tell approach. Important data visualization can benefit from this presentation technique. This is a specific strategy that can work for some but not all. The overarching principle is that an effective talk balances text with visuals and oral explanation. One must provide enough to read but not overwhelm so as to avoid the audience hurriedly reading throughout the presentation. Better they pay attention to you than to your slide deck.

## Rule 4: Use simple visuals

Slides advance very rapidly in these talk formats. Similar to the rules for better figures [15], identify the key message and avoid superfluous visual elements. Do not cut and paste figures prepared for written papers because the risk of losing the audience in a rapid talk is too great if they are expected to search, parse, or mentally rotate elements such as labels. Simplify data visualizations as needed and use color to show groupings and patterns. Visual guides and color are allowed here and not necessarily bound by the same rules as papers. Explicitly direct the audience to the key attribute of the visual you wish to highlight because there is no time for them to search for this visual point on each slide. Furthermore, if you choose to let the audience search on some slides, limit the number of slides that require more than cursory processing to one. For instance, use a single, relatively more complex visual slide to present the key figure showing the major quantitative finding of the study. A planned pause from rapid speaking is a powerful technique for the audience to catch their collective breath and also absorb this slide. Expecting an audience to do this 20 times in short order is unreasonable, and they will tune out. Use a separate slide to state the significance or interpretation for this finding and then begin speaking anew.

## Rule 5: Develop a consistent theme

In style, graphical design, language, and imagery, be consistent. This will ensure that the audience can allocate processing and scanning time on each slide to the salient elements that change and not to those that do not explain, support the science, or advance the main purpose of the presentation. The “branding” of your presentation and scientific message is important [3]. Use this consistency to reinforce the importance of your brand (and thus indirectly your message). Do not develop your brand using canned templates. These templates can be attractive but generally do not support the specifics of your talk and are often superfluous decoration.

## Rule 6: Repeat critical messages twice using different visuals

It is very easy to miss the main message in a rapid-fire talk, even more so than in a more traditional presentation. A total of 15 to 20 seconds to summarize the major implication or finding in a single slide is very short. Consider using a visual analog, metaphor, or simpler restatement of the major finding/implication in a subsequent slide. Typically, the assumption in these formats is that you do not cut and paste the exact same slide twice to provide oneself with more time (i.e., cheating), but you can certainly use a new slide to re-emphasize or extend the major finding. Three is a crowd and feels unduly repetitive in brief presentations. Stick with only one repetition.

## Rule 7: Use the principle of parsimony in explanations

Exactitude is as virtuous in literature as in science [13]. Identify concepts that require explanation and those that do not. Then, use simple explanations. Ensure the process or finding genuinely requires that explanation. Showing a finding and limiting what you say (Rule 4 for visuals in particular) can be a powerful means to emphasize importance. This technique also has the added benefit of providing the audience with the “space” to think, even momentarily, without distraction from the ongoing speaker dialogue. Some processes and patterns require little to no explanation. Use exactly that much. Statistics, field sampling, experimental design, and implementation strategy for the process proposed should be described in at most two succinct sentences within a 15- to 20-second interval. Explain what you need and consider engagement through less, not more, on some slides within the presentation deck.

## Rule 8: Allocate more than one slide to effectively end the narrative

At slides 16–17 in a 20-slide deck, begin closing the larger (and singular) story arc. Abrupt termination of a talk can be an effective means to jar or shock the audience but should be used sparingly—if ever. This technique comes at the cost of potential acceptance and reconciliation with the methods and implications offered. Do not leave the audience hanging. It is also natural for the audience to match the pacing and tempo of the speaker cognitively, and an abrupt end unnecessarily signals the end of a discussion and dialog.

## Rule 9: Use the final slide for contact information and links to additional resources

The total presentation time is likely a third, or less, relative to most traditional oral papers at scientific conferences. Furthermore, many rapid-fire series do not provide time for questions or feedback at the end of each presentation. This slide should reference your social media accounts, email, and website. Leverage your broader corpus of work and ideas through these links and provide a point of contact for questions. Another trick of the trade is to publish the slide deck online and provide a link to the deck within the deck at the end of the presentation. The audience will thus have an opportunity to follow-up and review the slides at a more leisurely pace if they are so inclined. Acknowledge key support, inspirations, and collaborators.

## Rule 10: Use timed practice

Speaking rapidly and clearly is not necessarily a given, even for accomplished speakers. The advancement of the slides without the speaker is a necessary condition for many of these rapid formats. Practice with the timing set in your preferred application (i.e., with autoadvance enabled via transitions between slides). There is a goldilocks effect in the number of words spoken for these formats. Too little can be awkwardly disconcerting. Too much is always disastrous. Furthermore, each slide need not suffer from the same limitations. Some require more, others less (see Rule 7). Use these differences to your advantage, and the optimal extent of description per slide can only be discovered through timed practice. Effective practice should include many of the following general approaches: stand up, speak out loud, rehearse several times without text or notes, invite an audience, record it, experiment with planned pauses, and vary pace to account for nerves or delays on the actual day. For rapid-fire talks, another common strategy is to practice with a few less seconds allocated per slide to compensate for lags when projected, audience reactions, or your movement on the stage.

## Comments

Rules are meant to broken, but not all of them and not all at once. If you elect to violate some of the rules above (best treated as suggestions), you can captivate with a story, change tempo by saying less more slowly on some slides and more on others to convey urgency, and highlight complexity without overwhelming. The audience is also an important consideration in how strictly one should consider adhering to these or any other set of proposed simple rules for scientific communication. Public talks should emphasize implications and effectively end the narrative as proposed above, whilst presentations for a group of scientists can typically invoke parsimony for explanations more directly and use appropriately technical language. The simplicity and accessibility of visuals can also be tempered by audience, and in some instances, visuals can be used to provide an analogy versus providing direct evidence or data visualization. The goal of these specific talk formats is to synthesize a topic for all audiences without a major commitment of their time. If your topic and use/misuse of the above rules stimulates some discovery for your audience and they elect to pursue the topic in greater depth, then you have absolutely succeeded. An alternative goal in considering these simple rules and in using a brief format to communicate science is to promptly share your passion for your science. If nothing else, address the “why” of the science at hand and emphasize that science is always a celebration of process and discovery. Time is up!

## References

[1] Fischhoff B. The sciences of science communication. Proceedings of the National Academy of Sciences. 2013;110:14033–9. 10.1073/pnas.1213273110 PMC3752164 23942125

[2] Jucan MS, Jucan CN. The Power of Science Communication. Procedia—Social and Behavioral Sciences. 2014;149:461–6.

[3] Bik HM, Dove ADM, Goldstein MC, Helm RR, MacPherson R, Martini K, et al Ten Simple Rules for Effective Online Outreach. PLoS Comput Biol. 2015;11(4):e1003906 10.1371/journal.pcbi.1003906 25879439 PMC4399988

[4] Moser SC. Reflections on climate change communication research and practice in the second decade of the 21st century: what more is there to say? Wiley Interdisciplinary Reviews: Climate Change. 2016;7(3):345–69.

[5] Baron N. Escape from the Ivory Tower: A Guide to Making Your Science Matter. Washington, DC: Island Press; 2010.

[6] Bowater L, Yeoman K. Science Communication—A Practical Guide for Scientists. Oxford, UK: Wiley-Blackwell; 2013.

[7] Bickford D, Posa MRC, Qie L, Campos-Arceiz A, Kudavidanage EP. Science communication for biodiversity conservation. Biological Conservation. 2012;151(1):74–6. 10.1016/j.biocon.2011.12.016.

[8] Bultitude K. The Why and How of Science Communication In: Rosulek P, editor. Science Communication. Europe: Pilsen: European Commission; 2011.

[9] Greenhow C, Gleason B. Social scholarship: Reconsidering scholarly practices in the age of social media. British Journal of Educational Technology. 2014;45(3):392–402.

[10] Van Noorden R. Online collaboration: Scientists and the social network. Nature. 2014;512:126–9. 10.1038/512126a 25119221

[11] McClain C, Neeley L. A critical evaluation of science outreach via social media: its role and impact on scientists. F1000 Research. 2015;3:300. 10.12688/f1000research.5918.1 PMC4376169 25866620

[12] Valkenburg PM, Peter J, Walther JB. Media Effects: Theory and Research. Annual Review of Psychology. 2016;67(1):315–38. 10.1146/annurev-psych-122414-033608 26331344

[13] Calvino I. Six Memos for the Next Millennium. Cambridge, Massachusetts: Harvard University Press; 1988.

[14] Bourne PE. Ten Simple Rules for Making Good Oral Presentations. PLoS Comput Biol. 2007;3(4):e77 10.1371/journal.pcbi.0030077 17500596 PMC1857815

[15] Rougier NP, Droettboom M, Bourne PE. Ten Simple Rules for Better Figures. PLoS Comput Biol. 2014;10(9):e1003833 10.1371/journal.pcbi.1003833 25210732 PMC4161295

